# The impact of COVID-19 related lockdown measures on self-reported psychopathology and health-related quality of life in German adolescents

**DOI:** 10.1007/s00787-021-01843-1

**Published:** 2021-07-10

**Authors:** Julian Koenig, Elisabeth Kohls, Markus Moessner, Sophia Lustig, Stephanie Bauer, Katja Becker, Rainer Thomasius, Heike Eschenbeck, Silke Diestelkamp, Vera Gillé, Alisa Hiery, Christine Rummel-Kluge, Michael Kaess, The ProHEAD Consortium, Michael Kaess, Stephanie Bauer, Markus Moessner, Julian Koenig, Sabrina Bonnet, Stella Hammon, Sophia Lustig, Regina Richter, Katja Bertsch, Romuald Brunner, Johannes Feldhege, Christina Gallinat, Peter Parzer, Johanna Sander, Rainer Thomasius, Silke Diestelkamp, Anna-Lena Schulz, Christine Rummel-Kluge, Sabrina Baldofski, Elisabeth Kohls, Lina-Jolien Peter, Mandy Rogalla, Sarah-Lena Klemm, Heike Eschenbeck, Vera Gillé, Laya Lehner, Katja Becker, Alisa Hiery, Jennifer Karl, Hans Joachim Salize, Elke Voss, Steffen Luntz

**Affiliations:** 1 Section for Experimental Child and Adolescent Psychiatry, Department of Child and Adolescent Psychiatry, University Hospital Heidelberg, Heidelberg, Germany; 2 University Hospital of Child and Adolescent Psychiatry and Psychotherapy, University of Bern, Bern, Switzerland; 3 Department of Psychiatry and Psychotherapy, University Leipzig, Leipzig, Germany; 4 Center for Psychotherapy Research, University Hospital Heidelberg, Heidelberg, Germany; 5 Department of Child and Adolescent Psychiatry, University Hospital Heidelberg, Blumenstraße 8, 69115 Heidelberg, Germany; 6 Institute of Psychology, Heidelberg University, Heidelberg, Germany; 7 Department of Child and Adolescent Psychiatry, Psychosomatics and Psychotherapy, Philipps-University of Marburg, Marburg, Germany; 8 Center for Mind, Brain and Behavior (CMBB), University of Marburg and Justus Liebig University Giessen, Giessen, Germany; 9 German Center for Addiction Research in Childhood and Adolescence, University Hospital Hamburg-Eppendorf, Hamburg, Germany; 10 Department of Psychology, University of Education Schwäbisch Gmünd, Schwäbisch Gmünd, Germany

**Keywords:** COVID-19, Lockdown, Psychopathology, Adolescents, Germany

## Abstract

The impact of school-closings on adolescents’ mental health and well-being in the management of the ongoing COVID-19 pandemic is subject to ongoing public debate. Reliable data to inform a balanced discussion are limited. Drawing on a large ongoing multi-site project in Germany, we assessed differences in self-reported psychopathology in a matched convenience-sample of adolescents assessed pre- (November 26, 2018 to March 13, 2020; *n* = 324) and post the first lockdown (March 18, 2020 to August 29, 2020; *n* = 324) early 2020 in Germany. We found no evidence for an increase in emotional and behavioral problems, depression, thoughts of suicide or suicide attempts, eating disorder symptoms, or a decrease in general health-related quality of life. Reported suicide plans significantly decreased from 6.14 to 2.16%. Similarly, conduct problems decreased in the post-lockdown period. Family risk-factors did not moderate these findings. The influence of socioeconomic status on emotional and behavioral problems as well as depression decreased during the lockdown. Based on the present findings, the first school-closing in Germany had no immediate and severe impact on adolescents’ well-being. However, caution is warranted as our data covers a fairly small, affluent sample over a limited time-span and long-term consequences cannot be ruled out.

## Introduction

The impact of the COVID-19 pandemic on mental health has been broadly discussed since its beginning in early 2020. Of particular concern to health-care professionals and the public are the consequences of preventive measures in all-day life, including social distancing, homeschooling, home office, not being able to meet friends and other family members, as well as limited possibilities for sports and other leisure activities. Many of these restrictions seem to especially affect children and adolescents and potentially their mental well-being [1]. On March 22nd, 2020 across Germany social contacts were limited to one person outside the own household. All German states mandated school and kindergarten closures on March 23rd, 2020 and postponed academic semesters. Some states started reopening schools implementing preventive measures on April 23rd with considerable differences between states and schools. There was no regular school-routine since then. Political decision-making needs to balance potential downstream consequences of preventive measures such as school closings [2]. However, the current evidence to guide these decisions is limited, impeding a balanced debate. Although calls to safely reopen schools qucikly gained dominance in the public debate, there are considerable concerns regarding the importance of schools for amplified virus transmission [3]. Reliable data on the mental-health consequences of school closings are required to inform this discussion.

In a recently published systematic review (12 studies, *n* = 12,262), Nearchou and colleagues showed that the COVID-19 pandemic had an impact on youth mental health [4], illustrating, in particular, an increase in depression and anxiety in adolescent cohorts. However, all of the 12 included studies were of low or moderate methodological quality, resulting in a call for further high-quality research addressing mental health consequences of COVID-19 related lockdown measures in this important target group. A major shortcoming of existing studies is the absence of well-matched samples including pre- and post-lockdown assessments. Thus, while great initiatives such as the Co-SPACE and Co-SPYCE studies have been initiated since the break-out of the pandemic, and child and adolescent mental-health receives considerable attention by now—methodological limitations and potential bias compromise the debate. Further, while COVID-19 and related lockdown measures might represent a general risk factor for elevated mental health problems (also see [5]), other studies have illustrated the complexity of these associations. For instance, depending on personal motives to socially distance, some adolescents actually reported less anxiety and depressive symptoms [6]. These inconsistent findings illustrate the important role of third factor variables, previously not accounted for, and the need for better data.

Family socioeconomic status (SES) and psychosocial risk factors (e.g. mental or chronic disease in one parent, growing up with a single parent, poverty, unemployment) may be such important third factors. Independent of the ongoing pandemic, both have been associated with mental health problems in adolescents [7, 8]. School-closings and the stay-at-home orders might have amplified their moderating influence, as these factors are often related to relatively cramped living conditions, no access to the outdoors, low family functioning and a resulting need for external (social) support in affected youth. While data from Germany are missing, such an idea is in line with reports from the UK, illustrating that one-third of households had at least one major housing problem related to overcrowding, affordability or poor-quality housing, potentially affecting health outcomes [9]. Housing has been identified as a determinant of COVID-19 inequities [10].

In the present study, we aimed to (a) assess the impact of COVID-19 related lockdown measures on adolescent mental health and (b) investigate the impact of SES and family risk-factors on these associations. We draw on matched pre- and post-lockdown data from a large ongoing multi-site project in Germany (“Promoting Help-seeking using E-technology for Adolescents with mental health problems” ProHEAD [11]), offering the unique opportunity to address the aforementioned aims and overcome limitations of previous studies. Importantly, the present study was initiated and first assessments took place (November 2018) before COVID-19 was raising awareness. Unlike other studies that were initiated to explicitly address the impact of COVID-19 on mental-health, ProHEAD data allow for an analysis of time-trends and changes in self-reports obtained from youth, minimizing bias.

## Methods

### General procedures

Data for the present analyses were taken from the ongoing ProHEAD project. ProHEAD is a multi-center consortium situated at five study sites across Germany and led by the managing site at the University Hospital of Heidelberg [11]. ProHEAD is an ongoing study with no previous intermediate data analysis. However, in the light of the ongoing pandemic, we decided for data release to address the questions at hand. The study protocol was approved by the Ethics Committee of the Medical Faculty at the University of Heidelberg (Study ID: S-086/2018) and subsequently at all involved study sites. In brief, ProHEAD aims to conduct longitudinal assessments of mental health problems in a sample of 15,000 children and adolescents aged ≥ 12 years. Following the completion of a computerized screening assessment, participants receive feedback on their individual results along with an invitation to register for one out of five clinical trials [11], not further detailed here. All screening assessments were conducted at participating schools during the pre-lockdown period. In the post-lockdown period, 63% of pupils completed their assessments at home. The general objective of ProHEAD is to investigate the efficiency and cost-effectiveness of different online interventions in the treatment and prevention of mental-health problems in those with mental-health problems or those at-risk for mental-health problems, as well as the promotion of mental-health in those without mental-health problems. The present cross-sectional analyses are based on data obtained within the screening assessment, following a data release of the ongoing project in early September 2020. Thus, data collected between the start of recruitment (November 2018) and August 2020 were included. From a total of *N* = 5408 completed assessments within this time-frame (*n* = 5084 pre-lockdown; *n* = 324 post-lockdown), a matched sample was drawn, reflecting assessments before (termed: pre-lockdown) the closing of schools in Germany (March 16, 2020) and thereafter (termed: post-lockdown). All available post-lockdown data were used and accordingly a matched pre-lockdown sample was drawn. Subjects were matched on age, sex and type of school using the “MatchIT” package, as implemented in R [12], resulting in a sample of *n* = 648 adolescents (50% each pre- and post-lockdown). Importantly, while ProHEAD implemented measure to recruit a representative sample of German youth, here we relied on a matched-convenience sub-sample of data collected until now.

### Instruments

The Strengths and Difficulties Questionnaire (SDQ) was used to measure emotional and behavioral problems [13]. The SDQ is a 25-item self-report instrument for children and adolescents between 11 and 17 years of age (SDQ-S11-17). Each item is rated on a 3-point scale. Five items each cover one of five sub-scales, concerning emotional problems, conduct problems, hyperactivity, peer problems and prosocial behavior. Studies in population-based samples suggest good psychometric properties. Here we used the SDQ sum score to index general psychopathological distress within the past 6 months. Alongside the SDQ, the 9-item version of the Patient Health Questionnaire (PHQ-9) modified for Adolescents (PHQ-A [14] was used to specifically assess depressive symptoms. The PHQ-A rates the frequency of depressive symptoms, resulting in a severity index, showing good psychometric properties [15]. The PHQ-A covers depressive symptoms within the past two weeks. Current eating disorder symptoms were assessed using the Weight Concerns Scale (WCS) and the Eating Disorder Examination-Questionnaire (EDE-Q). The WCS is a widely used 5-item measure assessing general risk factors for eating disorders, which has demonstrated its predictive value in prospective studies [16]. The EDE-Q is a self-report questionnaire with sound psychometric properties, its global scale consists of 23 items and is commonly used to assess eating disorder severity [17]. Health-related quality of life (HRQoL) was assessed using the German 10-item self-report version of the KIDSCREEN (KS-10) generic HRQoL measure for children and adolescents (8–18 years of age) [18]. The KS-10 is an international cross culturally comparable quality of life assessment instrument tailored for children and adolescents. The KS-10 index was used to index global HRQoL in the past seven days. Further, suicidal thoughts and behavior were assessed using the Paykel Suicide Scale (PSS; [19]). We selected three items to assess the 2-week prevalence of suicidal thoughts and behavior (active thoughts of taking one’s life, seriously considered taking one’s life, and attempted suicide). Each item was rated in a dichotomous fashion (yes/no). Concerning suicidality and participant safety, stopping rules for children and adolescents participating in the trial are the reporting of acute suicide plans or suicide attempts while participating in the ProHEAD intervention, as communicated with the case manager. In case of the reporting of acute suicide plans or attempts, special emergency procedures are put in place that allow immediate contact with the participant to assess risks and refer to appropriate care. Alongside sociodemographic confounds of sex and age, psychosocial risk factors, as well as socio-economic status, were assessed using the Laucht-Index [20] and the Family Affluence Scale (FAS [21]). The Laucht-Index comprises 10-items assessing potential family risk factors (e.g. unemployment). It distinguishes no risk (index score 0), low risk (index score 1 or 2) and high risk (index score > 2). The FAS is a 4-item self-report of family wealth, distinguishing between low FAS (scores 0–2); medium FAS (scores 3–5); and high FAS (scores 6–9), based on the FAS sum score.

### Statistical analyses

In a first step, chi-square tests and t-tests were used alongside descriptive statistics, to compare pre- and post-lockdown samples. Second, differences on clinical variables of interest (SDQ, PHQ-A, WCS, EDE-Q, KS-10, thoughts of suicide, suicide plans, attempted suicide) between the pre- and post-lockdown samples were assessed using linear (SDQ, PHQ-A, KS-10, WCS, EDE-Q) or logistic (thoughts of suicide, suicide plans, attempted suicide) regression analyses, with the dummy coded (0/1) grouping variable lockdown. Subsequently, the respective models were adjusted for additional main effects of sex, age, and in addition SES (continuous FAS score) and family risk factors (continuous Laucht score). Third, potential interactions between sex, age, as well as risk-factors (SES and Laucht score) and lockdown on clinical variables of interest were assessed. Finally, we used change-point analyses [22] implemented in R using the “changepoint” package [23] to assess changes in continuously scored measures of clinical interest (SDQ, PHQ-A, KS-10, WCS, EDE-Q) over time. A minimum of 10% subsequent cases (*n* = 32) were considered as a segment. Continuous measures (SDQ, PHQ-A, KS-10, WCS, EDE-Q) were *z*-standardized for linear-regression analyses, to enable better comparison of coefficients. All statistical calculations were performed using Stata/SE (16.0, Stata Corp LLC, College Station, TX, USA) at an alpha level of 0.05.

## Results

The pre-lockdown and post-lockdown samples each comprised n = 324 matched adolescents. Sociodemographic and clinical characteristics are provided in Table 1, indicating the affluent nature of the sample. Simple comparisons showed no significant differences between groups on any selected outcome of interest, with the exception of suicide plans (*χ*^2^_(1)_ = 7.316, *p* = 0.007) and the SDQ subscale of conduct problems (*t*_(646)_ = 2.005*, p* = 0.045). Reporting of suicide plans was significantly decreased (OR: 0.32) in the post-lockdown period (*n* = 7, 2.16%) compared to the pre-lockdown period (*n* = 21, 6.48%). Similarly, conduct problems decreased in the post-lockdown period.

**Table 1:** Sociodemographic and clinical characteristics by sample

	Pre-lockdown	Post-lockdown	*p*
*N* (female), *n*	324 (224)	324 (225)	Matched for
Age, mean (SD)	14.93 (1.88)	14.93 (1.88)	Matched for
Median, range [min–max]	15.00 [12.00–20.00]	15.00 [12.00–20.00]	
Participant born in Germany, *n* (%)	310 (95.68)	310 (95.68)	1
Father born in Germany, *n* (%)	238 (73.46)	256 (79.01)	0.186
Unknown, *n* (%)	3 (0.93)	1 (0.31)	
Mother born in Germany, *n* (%)	239 (73.77)	258 (79.63)	0.204
Unknown, *n* (%)	8 (2.47)	7 (2.16)	
School type, *n* (%)			Matched for
Oberschule and Gymnasium	162 (50.00)	163 (50.31)	
Realschule	25 (7.72)	25 (7.72)	
Haupt- & Werkrealschulen	25 (7.72)	24 (7.41)	
Gemeinschaftsschulen & Stadtteilschulen	112 (34.57)	112 (34.57)	
SDQ total, mean (SD)	12.36 (5.39)	11.98 (5.03)	0.351
Median, range [min–max]	12.00 [0.00–27.00]	12.00 [1.00–26.00]	
SDQ emotional, mean (SD)	4.56 (2.62)	4.00 (2.55)	0.214
Median, range [min–max]	4.00 [0.00–10.00]	4.00 [0.00–10.00]	
SDQ conduct, mean (SD)	2.00 (1.60)	1.76 (1.45)	0.045
Median, range [min–max]	2.00 [0.00–10.00]	2.00 [0.00–8.00]	
SDQ hyper, mean (SD)	3.51 (2.22)	3.53 (1.99)	0.911
Median, range [min–max]	3.00 [0.00–10.00]	3.00 [0.00–9.00]	
SDQ peer, mean (SD)	2.60 (1.66)	2.69 (1.62)	0.473
Median, range [min–max]	2.00 [0.00–9.00]	2.00 [0.00–8.00]	
SDQ social, mean (SD)	8.18 (1.67)	8.20 (1.59)	0.828
Median, range [min–max]	8.00 [3.00–10.00]	9.00 [1.00–10.00]	
PHQ-A, mean (SD)	7.95 (5.55)	7.39 (4.94)	0.169
Median, range [min–max]	6.5 [0.00–25.00]	6.00 [0.00–27.00]	
WCS, mean (SD)	31.67 (1.32)	30.56 (1.30)	0.550
Median, range [min–max]	26.67 [0.00–93.33]	26.67 [0.00–93.33]	
EDE-Q, mean (SD)	1.18 (0.07)	1.11 (0.07)	0.469
Median, range [min–max]	0.66 [0.00–5.45]	0.59 [0.00–5.68]	
KS-10, mean (SD)	27.64 (6.38)	27.34 (6.15)	0.539
Median, range [min–max]	28.00 [3.00–40.00]	28.00 [8.00–40.00]	
Thoughts of suicide, *n* (%)	44 (13.58)	33 (10.19)	0.182
Suicide plans, *n* (%)	21 (6.48)	7 (2.16)	0.007
Suicide attempts (%)	1 (0.31)	1 (0.31)	1
FAS, *n* (%)			0.701
Low	6 (1.85)	6 (1.85)	
Medium	79 (24.38)	70 (21.60)	
High	239 (73.77)	248 (76.54)	
Laucht-Index, *n* (%)			0.724
No risk	118 (36,42)	112 (34.57)	
Low risk	123 (37.96)	133 (41.05)	
High risk	83 (25.62)	79 (24.38)	
Laucht score, mean (SD)	1.54 (1.58)	1.64 (1.70)	0.431
Median, range [min–max]	1.00 [0.00–7.00]	1.00 [0.00–8.00]	
FAS score, mean (SD)	6.69 (1.81)	6.57 (1.70)	0.383
Median, range [min–max]	7.00 [2.00–9.00]	7.00 [1.00–9.00]	

School type: after 4 years of elementary school the German school system branches into three types of secondary schools. The so called *Haupt- & Werkrealschulen* (Secondary General School which takes 5 years after Primary School) prepares pupils for vocational training, whereas the *Realschule* (Intermediate Secondary School) concludes with a general certificate of secondary education after 6 years. Eight years of *Oberschule, Gymnasium* provide pupils with a general university entrance qualification; Gemeinschaftsschulen & Stadtteilschulen are secondary schools in Saxony

*SDQ* Strengths and Difficulties Questionnaire and respective sub-scales, *PHQ-A* Patient Health Questionnaire for Adolescents, *WCS* Weight Concerns Scales, *EDE-Q* Eating Disorder Examination-Questionnaire, *KS-10* KIDSCREEN (KS-10) generic HRQoL measure for children and adolescents, *FAS* Family Affluence Scale as index of socioeconomic status (SES), *Laucht-Index* assessing potential family risk factors

Linear and logistic regression analyses (Table 2) showed no additional significant main effects of lockdown in sex- and age-adjusted analyses. The respective coefficients and odds ratios (OR) indicated a slight decrease in psychopathological distress across measures in the post-lockdown period, not reaching statistical significance. The previously reported effect on suicide plans remained significant when adjusting the analyses for sex and age (*χ*^2^_(3)_ = 15.52, *p* = 0.001; OR: 0.31; 95% CI [0.13; 0.75]; *p* = 0.009). Similarly, the main effect on SDQ conduct problems was robust in sex- and age-adjusted analyses (*F*_(3;644)_ = 3.85, *p* = 0.010; lockdown (LD) coef: − 0.16; 95% CI [− 0.31; − 0.00]; *p* = 0.045). Similar, fully adjusted models, additionally controlling for family risk factor and SES, showed no additional main effect of lockdown, although the decrease in depression severity (PHQ-Q) neared statistical significance. Again, the effects observed on suicide plans (*χ*^2^_(5)_ = 32.82, *p* < 0.0001; OR: 0.27; 95% CI [0.11; 0.67]; *p* = 0.005) and conduct problems (*F*_(5;642)_ = 7.29, *p* < 0.0001; LD coef: − 0.17; 95% CI [− 0.32; − 0.02]; *p* = 0.026) remained significant also in fully-adjusted models.

**Table 2:** Results from regression models

Linear regression	Sex/age adjusted		Fully adjusted		Age inter	Sex inter	FAS inter	Risk inter
	*F*_3;644_ (*p*)	LD coef. (*p*)	*F*_5;642_ (*p*)	LD Coef. (*p*)	Interaction coef. (*p*)			
SDQ	8.69 (< .0001)	− 0.07 (0.334)	18.39 (< 0.0001)	− 0.10 (0.197)	− 0.00 (0.930)	0.06 (0.719)	0.10 (0.024)	− 0.03 (0.484)
SDQ: emotion	47.91 (< 0.0001)	− 0.10 (0.160)	37.90 (< 0.0001)	− 0.11 (0.100)	− 0.02 (0.514)	0.00 (1.00)	0.09 (0.031)	− 0.08 (0.064)
SDQ: conduct	3.85 (0.010)	− 0.16 (0.045)	7.29 (< 0.0001)	− 0.17 (0.026)	0.01 (0.792)	0.23 (0.171)	0.07 (0.112)	− 0.00 (0.918)
SDQ: hyper	1.82 (0.142)	0.01 (0.911)	4.90 (< 0.001)	− 0.00 (0.970)	− 0.01 (885)	− 0.05 (0.760)	0.01 (0.809)	0.03 (0.546)
SDQ: peer	1.45 (0.227)	0.06 (0.474)	6.97 (< 0.0001)	0.04 (0.596)	0.03 (0.548)	0.03 (0.829)	0.09 (0.045)	− 0.01 (0.854)
SDQ: social	8.53 (< 0.0001)	0.02 (0.836)	5.21 (< 0.001)	0.02 (0.826)	− 0.05 (0.245)	− 0.11 (0.529)	− 0.08 (0.060)	− 0.06 (0.192)
PHQ-A	22.26 (< 0.0001)	− 0.11 (0.144)	31.95 (< 0.0001)	− 0.13 (0.062)	− 0.01 (0.720)	0.10 (0.521)	0.09 (0.034)	− 0.08 (0.059)
WCS	26.11 (< 0.0001)	− 0.05 (0.510)	20.67 (< 0.0001)	− 0.06 (0.414)	− 0.06 (0.124)	− 0.03 (0.848)	0.05 (0.226)	− 0.07 (0.119)
EDE-Q	21.21 (< 0.0001)	− 0.06 (0.435)	21.35 (< 0.0001)	− 0.07 (0.321)	− 0.05 (0.181)	− 0.07 (0.663)	0.05 (0.189)	− 0.08 (0.061)
KS-10	27.00 (< 0.0001)	− 0.07 (0.529)	37.70 (< 0.0001)	− 0.02 (0.751)	0.04 (0.279)	0.01 (0.955)	− 0.07 (0.081)	0.04 (0.348)

Logistic regression	Sex/age adjusted		Fully adjusted		Age inter	Sex inter	FAS inter	Risk inter
	Chi^2^_3_ (*p*)	LD OR (*p*)	Chi^2^_5_ (*p*)	LD OR (p)	Interaction OR (*p*)			
Thoughts of suicide	16.77 (< 0.0001)	0.71 (0.173)	40.99 (< 0.0001)	0.67 (0.116)	1.00 (0.990)	2.46 (0.232)	1.15 (0.309)	0.91 (0.473)
Suicide plans	15.52 (< 0.0001)	0.31 (0.009)	32.82 (< 0.0001)	0.27 (0.005)	1.02 (0.922)	Ins. Obs	0.93 (0.753)	0.99 (0.956)
Suicide Attempts	0.34 (< 0.0001)	1.00 (0.998)	4.19 (0.523)	0.76 (0.852)	0.50 (0.376)	Ins. Obs	0.82 (0.789)	1.45 (0.618)

FAS and Laucht-Score were modelled as continuous variables in the respective regression analyses; SDQ (and sub-scales), PHQ-A, WCS, EDE-Q, and KS-10 scores were z-standardized to enable better comparison of coefficients; Ins. Obs.: insufficient observations; for better readability, results showing a significant effect of the lockdown are highlighted

*LD* lockdown, *OR* odds ratio, *fully adjusted* adjusted for age, sex, FAS, and Laucht-Score

**Fig. 1:**
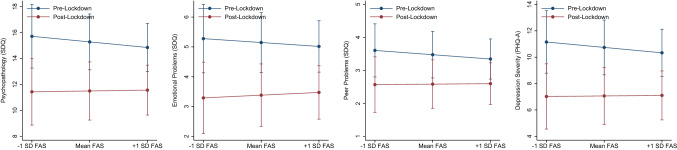
Interaction of socioeconomic status with lockdown in predicting general psychopathology (SDQ), emotional problems (SDQ), peer problems (SDQ) and depression severity (PHQ-A); for illustrative purposes mean family affluence (FAS) and ± 1 standard deviation (SD) were illustrated. SDQ and PHQ-A raw-scores are provided, respective models were based on *z*-standardized values for better comparison

With the exception of SES (FAS-score), analyses showed no significant interactions of potential confounders (age, sex, family risk) with lockdown in predicting psychopathological distress across measures. SES showed significant interactions with lockdown in predicting SDQ (*F*_(6;641)_ = 16.27, *p* < 0.0001; inter. coef. = 0.10; 95% CI [0.01; 0.18], *p* = 0.024), SDQ emotional problems (*F*_(6;641)_ = 32.54, *p* < 0.0001; inter. coef. = 0.09; 95% CI [0.01; 0.16], *p* = 0.031), SDQ peer problems (*F*_(6;641)_ = 6.51, *p* < 0.0001; inter. coef. = 0.09; 95% CI [0.00; 0.17], *p* = 0.045), and PHQ-A (*F*_(6;641)_ = 27.52, *p* < 0.0001; inter. coef. = 0.09; 95% CI [01; 0.16], *p* = 0.034). A graphical representation of these interactions is provided in Fig. 1. As illustrated, whereas during the pre-lockdown period, SES showed an association with the severity of psychopathological distress (greater distress in those with lower SES), this effect was diminished in the post-lockdown phase.

**Fig. 2:**
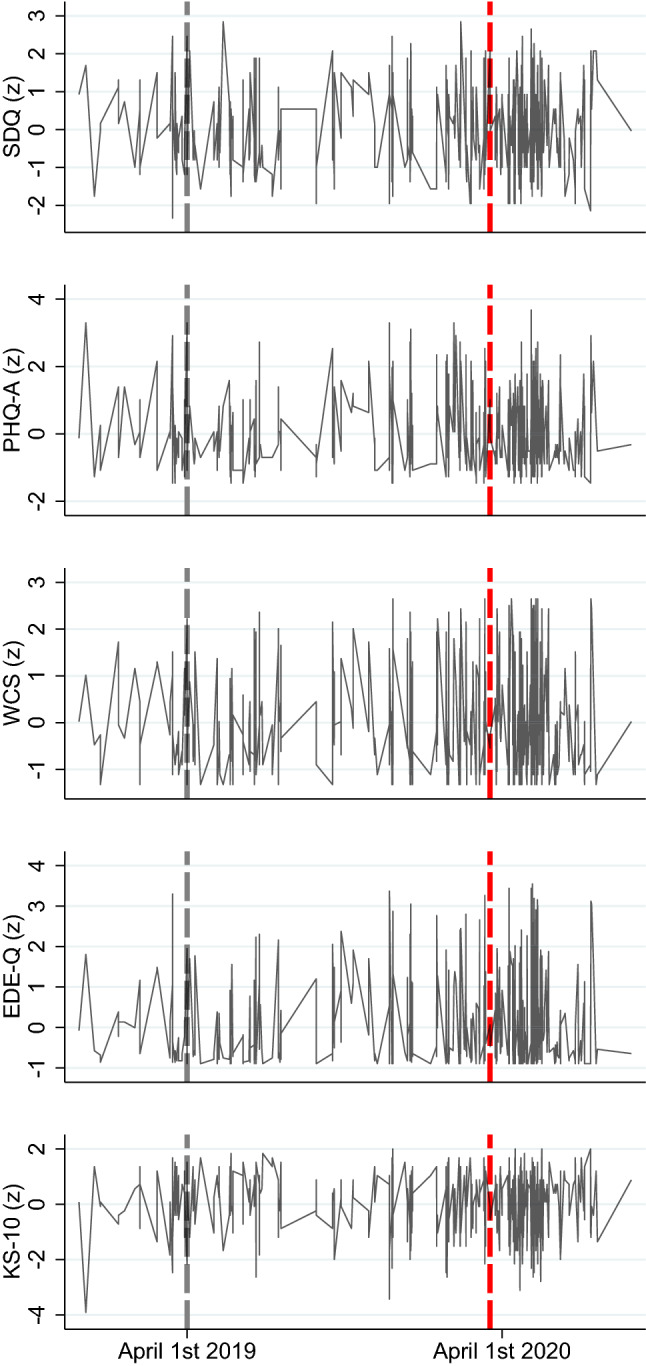
Data over time; displayed are the clinical outcomes (SDQ, PHQ-A, WCS, EDE-Q, and KS-10) by assessment time. Connected visualization for illustrative purposes, *SDQ* Strengths and Difficulties Questionnaire, *PHQ-A* Patient Health Questionnaire for Adolescents, *WCS* Weight Concerns Scales, *EDE-Q* Eating Disorder Examination-Questionnaire, *KS-10* KIDSCREEN (KS-10) generic HRQoL measure for children and adolescents. Red dashed line illustrates the date that lockdown measures were implemented (March 16, 2020); all measures *z*-standardized for better comparison

**Fig. 3:**
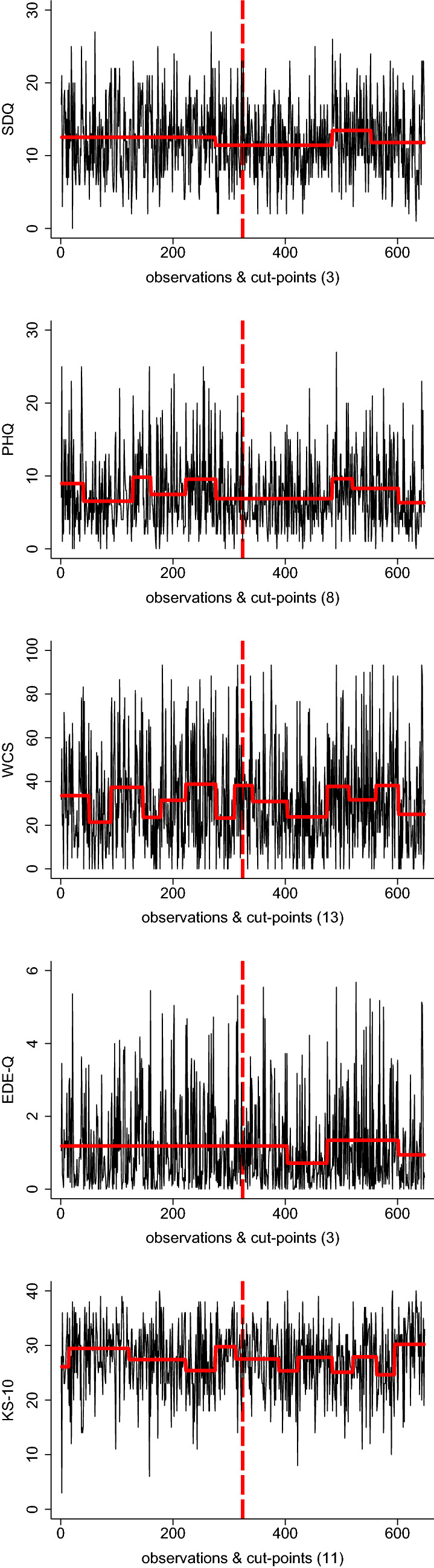
Change point analyses; displayed are the raw data of clinical outcomes (SDQ, PHQ-A, WCS, EDE-Q, and KS-10) by assessment time and corresponding segments differentiated by cut-points. Connected visualization for illustrative purposes. *SDQ* Strengths and Difficulties Questionnaire, *PHQ-A* Patient Health Questionnaire for Adolescents, *WCS* Weight Concerns Scales, *EDE-Q* Eating Disorder Examination-Questionnaire, *KS-10* KIDSCREEN (KS-10) generic HRQoL measure for children and adolescents. Red dashed line illustrates the date that lockdown measures were implemented (March 16, 2020); the number of cut-points is provided in brackets

Change point analyses were conducted independently on sample allocation to illustrate trends in data over time, as illustrated in Fig. 2. Analyses revealed a heterogeneous number of changes points, contributing to differences in test statistics for the different measures of interest, providing no evidence for a clear shift in symptom distress over time. The respective patterns were inconsistent and, in all cases, unrelated to the lockdown, as illustrated in Fig. 3.

## Discussion

Comparing relatively small pre- and post-lockdown samples of adolescents from an ongoing population-based study in Germany (ProHEAD), we found no statistically significant differences regarding emotional and behavioral problems (assessed with the SDQ), depression (PHQ-A), eating disorder symptoms (WCS and EDE-Q), quality of life (KS-10), as well as thoughts of suicide or suicide attempts. One finding showed statistical significance: reported suicide plans decreased substantially from 6.14 to 2.16%. However, given that suicide plans were reported only by a few subjects (*n* = 7 during the post-lockdown period and *n* = 21 during the pre-lockdown period), these findings should be interpreted with caution. In principal, findings from the present study contradict the widespread opinion and findings from other studies (e.g. the COPSY study) suggesting a potential increase in mental health problems in adolescents associated with COVID-19 related lockdown measures. Findings should inform the public debate, based on questionable evidence and (in many instances) overestimating the consequence of school-closings on mental health in youth.

Assessing specific dimensions of psychopathology, our findings do not support the general assumption of a deterioration of depression in children and adolescents [1]. While suicidality and suicide plans are fluctuating influenced by many risk factors [24], there is evidence for a relationship between suicidality and the school year calendar [25]. Thus, school closures may also have contributed to a decrease in immediate risk factors such as pressure for academic achievement [26] or school bullying [27]. We did not find any increases in eating disorder risk or impairment due to the lockdown, although a negative impact of the pandemic on individuals with eating disorders has been demonstrated in previous research [28]. Yet, in nonclinical samples like the one investigated in the present study, social comparison processes, weight and shape shaming, and teasing play an important role in the development of body dissatisfaction. It seems reasonable to assume that the harmful effects of these risk factors are decreased due to social isolation, attenuating the negative effects in the present sample.

Regarding the quality of life, our findings do not confirm data from Norway [29]. The authors of a cross-sectional study in dolescents (*N* = 2205) lower mean HRQoL as compared to European norms during the COVID-19 pandemic. Interestingly, however, Riiser and colleagues saw that being in quarantine/isolated and having suspected/confirmed COVID-19 was significantly associated with lower HRQoL, but seeing less friends than normal was not. Interestingly, third factors investigated in the present analyses (SES) lost their moderating influence on general psychopathology and depression during lockdown. Potentially, SES is of limited importance when money cannot be spent to enable leisure activities, or other immediate threats—such as a pandemic—dominate.

Overall, our findings suggest that during the first lockdown in March 2020 in Germany adolescents’ mental health problems did not considerably vary. While our data do appear comforting, they do not implicate that there is no need to support children and adolescents in a pandemic situation. Previously, we have described an increase in the utilization of the ProHEAD online interventions during the lockdown period in March 2020 [30]. Further, although our methodology allowed us to capture potential immediate effects of school-closings, it is well possible that actual effects on mental health are observed in longer follow-ups.^1^ Recent data from Japan show that suicide rates increased following an initial decline during the pandemic [32], illustrating a complex temporal association and potential long-term consequences. Importantly, alongside primary educational objectives, schools serve a considerable function in the early detection of mental health and family-related problems. Careful monitoring is required to fully understand the consequences of school-closings on different levels of observation.

Several limitations need to be acknowledged when discussing our findings. First, the present sample for analysis might not be representative for adolescents in Germany. Parents allowing their child to participate in a school-based study and those who are able to organize the parental consent etc. might not represent underprivileged households. As indicated, the sample was fairly educated and affluent. For a critical discussion on potential beneficial effects of lockdown-measures for some children and adolescents, also see [33, 34]. Importantly, as the available post-lockdown data built the reference for our matching procedure, the present sample might not align with the representativeness of the overall ProHEAD study. As matching was based on three variables only—that were likely skewed in the post-lockdown sample—findings might not generalize without limitations. As reported, most of the adolescents went to schools providing a general university entrance qualification. This is reflective of the German school system (e.g. based on data of the Federal Office of Statistics the majority (> 50%) of German youth in this age group attended Gymnasium in 2019/2020). Importantly, samples were matched for school type and testing for interactions with school type in exploratory analyses showed no robust effects. Still, the present findings might not generalize to more diverse samples. Second, we did not apply a within-subject design in a longitudinal manner, potentially enabling sampling bias. However, in contrast to previous studies [1], we were able to use matched pre- and post-lockdown samples. The median date of pre-lockdown data collection was November 25 2019 and May 5 2020 for the post-lockdown sample, respectively. Other population-based studies in Germany, such as the most recently published COPSY study [35], compared data assessed during a limited time within the pandemic—where restrictions were to a certain degree already lifted (here: May 26 to June 10, 2020)—with normative data collected several years earlier (here: 2017), thus potentially introducing bias. For clarity, assessments in the COPSY study were conducted in line with the pre-pandemic BELLA study, covering pre-pandemic data. Other great initiatives, such as the Co-SPACE and Co-SPYCE studies or respective WHO initiatives [36], do not include pre-pandemic assessments. Importantly, unlike others, the present study was initially not designed to assess COVID-19 related effects on youth mental-health. Thus, carrying no bias related to the respective assessments or recruitment of participants for a specific study purpose. We argue that recruiting subjects for participation in a specifically designed COVID-19 study is prone to introduce bias (e.g. sampling subjects with respective problems) when it comes to the reporting of mental-health problems.

The COVID-19 pandemic has an undisputed impact on all facets of our daily life. Although concerns for the mental health of adolescents are warranted, the public debate should be informed by reliable data. Based on the present findings, we see no evidence for a significant increase in mental health problems among youth at the time of the first school-closings within the first wave in Germany. Although we cannot draw causal conclusions, concerning the impact of respective political measures on adolescents’ mental-health and well-being, our data speak against any significant increase in mental-health problems at the time of interest. However, caution is warranted as our data cover a limited time-span only. As evidence is accumulating concerning the long-term consequences of long-lasting political measures on youth mental health, it is important to express clearly: here we only assessed short-lived consequences of the very first political measures in the management of the first wave of the pandemic. As researchers and clinicians, we are under the impression that there was a considerable increase in mental-health problems among youth within the second and third wave. This impression is further supported by the continuous release of data of the aforementioned studies, implemented to assess changes in the reporting of mental-health problems among youth during the pandemic. Future studies are needed to assess the long-term impact of the COVID-19 pandemic and associated political measures in its management during the second and third COVID-19 wave.

## Footnotes

1 A longer follow-up of the COPSY study has been published while the present manuscript was under review [31].
